# p110γ/δ Double-Deficiency Induces Eosinophilia and IgE Production but Protects from OVA-Induced Airway Inflammation

**DOI:** 10.1371/journal.pone.0159310

**Published:** 2016-07-21

**Authors:** Benedikt Mothes, Kirsten Bucher, Susanne Ammon-Treiber, Matthias Schwab, Roland P. Piekorz, Emilio Hirsch, Bernd Nürnberg, Sandra Beer-Hammer

**Affiliations:** 1 Department of Pharmacology and Experimental Therapy, Institute of Experimental and Clinical Pharmacology and Toxicology and ICePhA, University of Tuebingen, 72074 Tuebingen, Germany; 2 Department of Pharmacology, Toxicology and Clinical Pharmacy, Institute for Pharmacy, University of Tuebingen, 72076 Tuebingen, Germany; 3 Department of Clinical Pharmacology and ICePhA, University of Tuebingen, and Dr. Margarete Fischer-Bosch Institute of Clinical Pharmacology, 70376 Stuttgart, Germany; 4 Institutes for Biochemistry and Molekular Biology II, University of Duesseldorf, 40225 Duesseldorf, Germany; 5 Department of Genetics, Biology and Biochemistry, University of Torino, 10126 Torino, Italy; Research Center Borstel, GERMANY

## Abstract

The catalytical isoforms p110γ and p110δ of phosphatidylinositide 3-kinase γ (PI3Kγ) and PI3Kδ play an important role in the pathogenesis of asthma. Two key elements in allergic asthma are increased levels of eosinophils and IgE. Dual pharmacological inhibition of p110γ and p110δ reduces asthma-associated eosinophilic lung infiltration and ameliorates disease symptoms, whereas the absence of enzymatic activity in p110γ^KO^δ^D910A^ mice increases IgE and basal eosinophil counts. This suggests that long-term inhibition of p110γ and p110δ might exacerbate asthma. Here, we analysed mice genetically deficient for both catalytical subunits (p110γ/δ^-/-^) and determined basal IgE and eosinophil levels and the immune response to ovalbumin-induced asthma. Serum concentrations of IgE, IL-5 and eosinophil numbers were significantly increased in p110γ/δ^-/-^ mice compared to single knock-out and wildtype mice. However, p110γ/δ^-/-^ mice were protected against OVA-induced infiltration of eosinophils, neutrophils, T and B cells into lung tissue and bronchoalveolar space. Moreover, p110γ/δ^-/-^ mice, but not single knock-out mice, showed a reduced bronchial hyperresponsiveness. We conclude that increased levels of eosinophils and IgE in p110γ/δ^-/-^ mice do not abolish the protective effect of p110γ/δ-deficiency against OVA-induced allergic airway inflammation.

## Introduction

Asthma is a chronic inflammatory syndrome of the airways [1], which affects about 241 million people worldwide [2]. The most common manifestation of this syndrome is the allergic asthmatic phenotype [3]. It is characterized by mucus hypersecretion and hyperplasia of lung tissue causing breathlessness, cough and wheezing. Moreover, it is accompanied by an elevated bronchial hyperresponsiveness [4]. A key immunological feature of allergic asthma is a Th2 cell-driven eosinophilia in the lung and blood, accompanied by elevated IgE levels [5]. It has been shown that eosinophils are required for the development of experimental allergic asthma in C57BL/6 mice [6,7] and the severity of asthma was correlated with increasing eosinophil levels in human patients [8]. Allergen-specific IgE plays a major role in the development of chronic airway inflammation [9]. Monomeric IgE has been shown to promote mast cell survival and cytokine secretion in the absence of cross-linking by allergens [9,10].

Various studies demonstrate that phosphatidylinositide 3-kinase γ (PI3Kγ) and δ (PI3Kδ) play a central role in the pathogenesis of allergic asthma and contribute to eosinophilic inflammation and bronchial hyperresponsiveness [11–14]. PI3Kγ and PI3Kδ lipid kinases are heterodimers formed by a catalytic and a regulatory subunit. The two isoforms belong to the PI3K class I family and are activated through partially overlapping pathways [15]. PI3Kγ is mainly stimulated by G protein-coupled receptors (GPCRs), such as chemokine receptors, through interaction with Gβγ dimers and Ras, whereas PI3Kδ is predominantly responsive to receptor tyrosine kinases, Toll-like receptors, cytokine and antigen receptors, and also Ras [15,16].

The catalytic PI3K subunits p110γ and p110δ are mainly expressed in hematopoietic cells, where they control various functions, including proliferation, differentiation, migration and survival of leukocytes [17]. While p110γ is involved in the development of T cells [18], growth factor-induced anti-apoptotic signalling in neutrophils [19], and the migration of macrophages [20], neutrophils [16,20] and eosinophils [12,13,21], p110δ is required for the differentiation and recruitment of Th cell subsets [22–24] and the development of B cells [25,26].

Pharmacological inhibitors of PI3Kγ/δ have been investigated for the treatment of inflammatory diseases [27,28]. TG100-115, a dual PI3Kγ/δ inhibitor, showed beneficial effects on the development and progression of eosinophilic asthma and neutrophilic-driven COPD in mice [28]. Treatment with IPI-145, another dual PI3Kγ/δ inhibitor, resulted in a significant reduction of allergic symptoms in an ovalbumin (OVA)-induced asthma model in rats [27]. While these studies indicate that compounds inhibiting both PI3Kγ and δ may be effective in the treatment of asthma, analyses of mice lacking PI3Kγ/δ enzymatic activity suggest that long-term inhibition during treatment with dual inhibitors harbours potential risks. Indeed, we and others have shown that p110γ/δ double knock-out (KO) (p110γ/δ^-/-^) mice suffer from severe immune defects, including B cell lymphopenia [29] and T cell lymphopenia [30], the latter of which has also been observed after administration of the dual-specific pharmacological p110γ/δ inhibitor CAL-130 [31]. Lymphopenia and a restricted T cell reservoir are often associated with a Th2-driven eosinophilia in humans [32] and mice [33]. Interestingly, in mice lacking p110γ and expressing a catalytically inactive p110δ isoform (p110γ^KO^/δ^D910A^ mice), lymphopenia is indeed accompanied by infiltration of mucosal tissues by eosinophils, and hyperimmunoglobulinemia IgE [23]. Eosinophils and IgE are critically involved in the pathogenesis of allergic asthma. Therefore, eosinophils increased by a permanent block in p110γ/δ signalling might counteract the protective effect of dual p110γ/δ inhibition on the onset and progression of asthma.

To test this hypothesis, we examined the immune response of p110γ/δ^-/-^ mice in an OVA-induced allergic airway inflammation model. We determined (1) the distribution of eosinophils, concentrations of the corresponding hematopoietic growth factor IL-5, and IgE levels in a basal state, and (2) the immune response in the OVA-induced airway inflammation model. Despite a pronounced eosinophilia and hyperimmunoglobulineamia IgE, the lack of both p110 kinases in p110γ/δ^-/-^ mice protected against OVA-induced allergic airway inflammation and bronchial hyperresponsiveness.

## Materials and Methods

### Animals

Generation of p110γ^-/-^, p110δ^-/-^ and p110γ/δ^-/-^ mice was described previously [29]. Mice used in this study were on a C57BL/6N genetic background (Charles River). For all experiments 8–14 week-old male mice were used.

### Ethics statement

Animal experiments were conducted in accordance with the recommendations in the Guide for the Care and Use of Laboratory Animals (FELASA). Animals were kept in standard cages with enrichment under specific pathogen-free conditions (SPF) according to national guidelines for animal care at the animal facility of the University of Tuebingen. All animals were kept with a 12 hour light/dark cycle and had access to food and water *ad libitum*. The physical condition was monitored daily both before and during the experiments. No animal became severely ill or died before the experimental endpoint. Protocols were approved by the committee on the Ethics of Animal Experiments of local authority “Regierungspräsidium Tuebingen” (permit number: PH4/11, PH3/12 and §4 Mitteilung vom 26.11.2009 and 6.12.2013). All surgery was performed under anaesthesia and all efforts were made to minimize suffering. For IPL and BALF mice were anaesthetized with Pentobarbital (120 mg/kg BW).

### Ovalbumin-dependent induction of allergic airway inflammation

An ovalbumin (OVA)-dependent allergic airway inflammation was induced as described previously [14,34] and illustrated in S1 Fig. In short, mice received a 200 μl i.p. injection on day 1 and 14 containing 20 μg OVA (Sigma-Aldrich) and 1 mg of Imject^TM^ Alum (Thermo Scientific). Subsequently, all mice were challenged for 30 minutes on days 21, 22 and 23 with an aerosolised OVA solution (3% OVA in DPBS (both from Sigma-Aldrich)) or DPBS alone for the control group. For the challenge, animals were placed in a Plexiglas chamber and the aerosol with a median mass diameter of 2.2 μM was generated with a PARI BOY SX (Pari). On day 25, animals were euthanised with 120 mg/kg of sodium pentobarbital (Sigma-Aldrich), and were either used to determine airway resistance in the isolated perfused lung (IPL) model or to analyse leukocyte infiltration into the bronchoalveolar space and the lung tissue.

### Preparation of leukocyte suspensions from spleen, blood, bone marrow, lung tissue and BALF

Leukocyte suspensions from spleen, blood, bone marrow (BM) and lung tissue were isolated as described previously [29,35]. To isolate leukocytes from bronchoalveolar lavage fluid (BALF) a bronchoalveolar lavage (BAL) was performed. To this end euthanised animals were tracheotomised and the trachea cannulated. Then, mice were exsanguinated and BAL was performed by instillation of 400 μl ice cold PBS for four times. BALF was collected and BALF cells were isolated by centrifugation at 500 x g for 5 min.

### Cellspin preparation

Cellspins were performed on silan coated specimen holders with the Cellspin II (Tharmac) at 6°C (75 x g, ramp 4, break 6) for 10 min. 1/5 of cells collected by BAL were used for cellspin preparation.

### Flow cytometry

For flow cytometric characterisation of leukocytes from spleen, blood, BM, lung tissue, and BALF the following antibodies were used: F4/80 FITC (1:100, AB_893500, clone BM8; BioLegend), CD3ε Pacific Blue (1:100, AB_397063, clone 500A2), CD3ε PerCPCy5.5 (1:100, AB_10562558, clone 145-2C11), CD11b PE-Cy7 (1:400, AB_394491, clone M1/70), CD19 V450 (1:100, AB_1645269, clone 1D3), Ly6G FITC (1:100, AB_10562567, clone 1A8), Siglec-F PE (1:200, AB_394341, clone E50-2440; all BD Bioscience), Ly6G APC (1:1600, 17-9668-82, clone 1A8; eBioscience). Flow cytometry was performed with the FACS Canto II (BD Bioscience) and the obtained data were analysed using FlowJo 7.6.1 (FlowJo).

### Determination of airway resistance

Airway resistance in response to methacholine (MCh, acetyl-β-methylcholine chloride; Sigma-Aldrich) was determined with the *ex vivo* model of the IPL (Harvard Apparatus) [34]. In brief, *in situ* mouse lungs were placed in a heated (38°C) thorax chamber and mice were ventilated *via* a tracheal cannula. Ventilation rate was set to 90 breaths per minute with negative pressure ventilation between -2.7 cm H_2_O and -8.5 cm H_2_O. To prevent atelectasis, a hyperinflation was triggered every 5 minutes (-25 cm H_2_O). Lungs were perfused with a buffer containing 4% hydroxyethyl starch (Serumwerk Bernburg) *via* the pulmonary artery at a flow rate of 1 ml/min. After a 20 minute baseline measurement, lungs were perfused with increasing concentrations of MCh (5 μM, 50 μM and 500 μM) in perfusion buffer for 10 minutes each. Between concentrations lungs were washed with perfusion buffer only for 20 min. Physiological lung parameters, including airway resistance, were recorded automatically and analysed by HSE-HA Pulmodyn W Software 1.1.1.202 (Harvard Apparatus). For statistical and graphical analysis, the mean resistance values were calculated from the last 10 time stamps (40 seconds) starting 5 min after each 10 minute of MCh exposure.

### Measurement of cytokines, total and OVA-specific IgE

Serum concentrations of IL-5 in peripheral blood were determined with a Bio-Plex mouse cytokine 23-plex assay (Bio-Rad Laboratories), according to the manufacturer's protocol. In brief, the bead suspension was incubated with standard, samples, or blank for 30 min under continuous shaking in a 96-well filter plate. Then, the plate was washed three times. Beads were resuspended in biotinylated detection antibody solution and incubated for 30 min. After three washing steps, streptavidin-phycoerythrin was added and the plate was incubated for 10 min. After three washing cycles, the plate was analysed using a Bio-Plex 200 suspension array system (Bio-Rad Laboratories).

Serum concentrations of total IgE were determined using an IgE ELISA kit (BD Bioscience). Measurements were performed according to the manufacturer’s protocol. In brief, half-area plates (Greiner Bio-One) were coated over night with anti-IgE capture antibody. After blocking the plates with 10% FBS (Gibco^®^ Life Technologies) in DPBS, samples were incubated together with an IgE standard. After 2 hours, detection antibody coupled with streptavidin-horseradish peroxidase was added. 3,3’,5,5’-Tetramethylbenzidine (TMB; Thermo Scientific) was used as substrate and IgE concentration was determined with a Tecan sunrise reader (Tecan). To detect OVA-specific IgE, the identical kit was applied with the following modifications, i.e. plates were coated with 20 μg/ml OVA and OVA-specific IgE (top standard 100ng/ml, clone 2C6, AB_2285753, AbD Serotec^®^) was used as standard.

### Histochemistry

Left lungs were collected after IPL and fixed with Roti^®^-Histofix 4% (Carl Roth) for 72 hrs and then embedded in paraffin for microsections. Slices of 4–6 μm were deparaffinised and rehydrated before staining with a PAS staining kit (Carl Roth) according to the manufacturer’s protocol. Stained tissue was analysed with a Zeiss Axio Image M2 microscope (Zeiss). PAS-positive goblet cells were counted at 200x magnifications and were quantified per 1 mm of basement membrane.

### Statistical analysis

Statistical analyses were performed as indicated in the figure legends. All calculations were performed using GraphPad Prism 5.01 (GraphPad Software). A value of p ≤ 0.05 was considered statistically significant.

## Results

### p110γ/δ deficiency increases eosinophils, IL-5 and IgE

Eosinophil-derived mediators and IgE are major contributors to allergic asthma [5,9]. Although dual inhibitors of PI3Kγ and δ are considered as promising agents in the treatment of asthma, mice constitutively lacking PI3Kγ and -δ enzymatic activity suffer from eosinophilic inflammation in mucosal tissues and IgE hyperproduction [23], suggesting that pharmacological long-term inhibition of PI3Kγ and -δ may eventually aggravate asthma by inducing eosinophilia. Here, we examined p110γ/δ double KO mice (p110γ/δ^-/-^ mice) and determined their basal eosinophilic response and its potential influence on the development of OVA-induced allergic airway inflammation.

**Fig 1:**
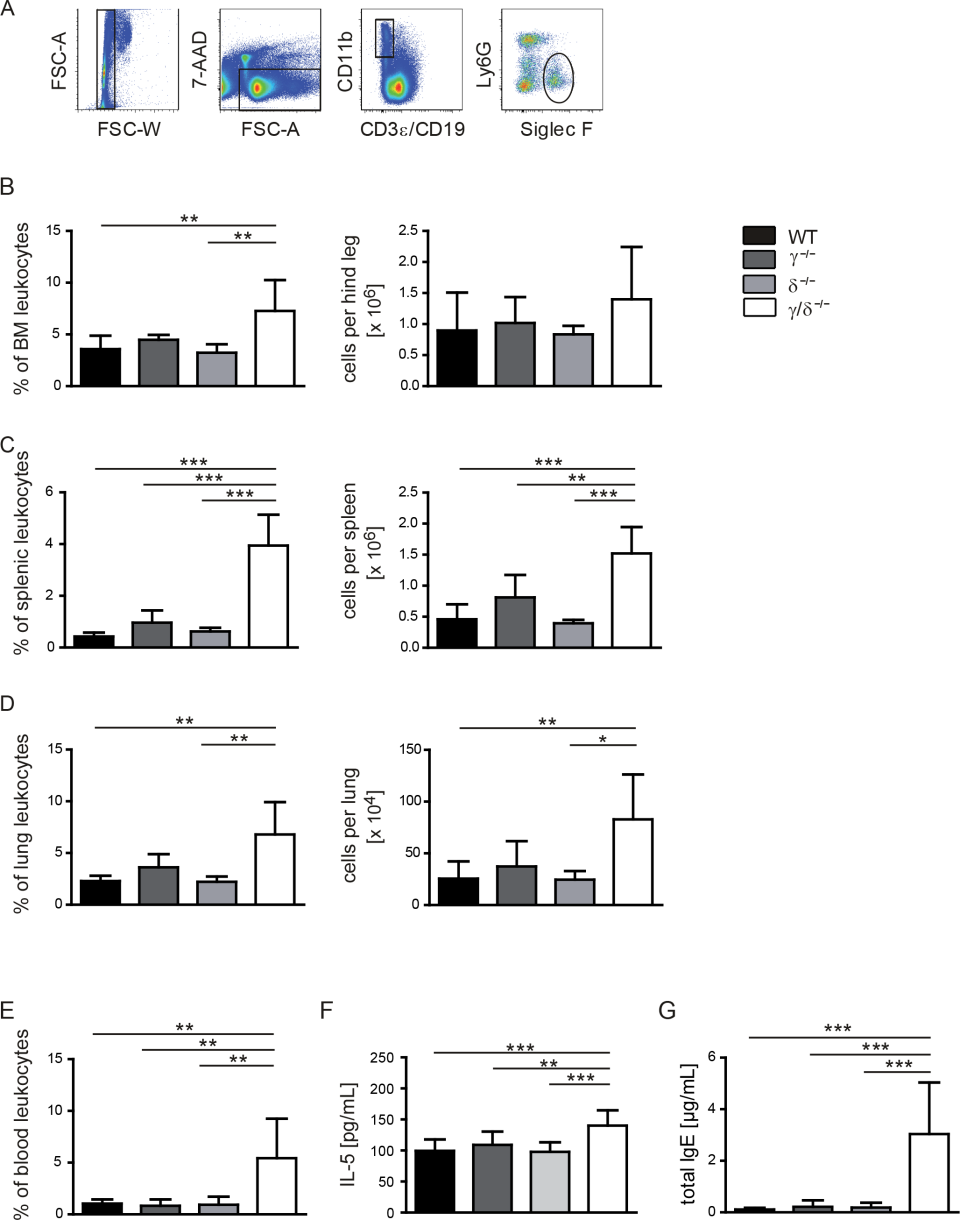
Numbers of eosinophils and serum concentrations of IL-5 and IgE are elevated in p110γ/δ^-/-^ mice. (**A—E**) Eosinophil numbers in p110γ^-/-^, p110δ^-/-^, and p110γ/δ^-/-^ and WT mice (n = 5–8). To determine eosinophil numbers, leukocyte suspensions from BM, spleen, lung, and blood were analysed by flow cytometry. (**A**) Eosinophils were gated as CD3ε^-^ CD19^-^ CD11b^+^ Ly6G^-^ Siglec F^+^ singlet leukocytes. Lung eosinophils were stained and gated as shown in S2 Fig. Percentages of living cells (FSC/SSC gate) and total cell counts of eosinophils (**B**) in the BM, (**C**) in the spleen, (**D**) in the lung, and (**E**) in the blood are depicted. Serum concentrations of (**F**) IL-5 and (**G**) IgE in p110γ^-/-^, p110δ^-/-^, p110γ/δ^-/-^ and WT mice (n = 10). Bars represent means + SD. Data were analysed by One-way ANOVA followed by Bonferroni’s comparison tests for selected pairs of columns. ***P < 0.001, **P < 0.01, *P < 0.05. Asterisks indicate differences in comparison to p110γ/δ^-/-^ mice.

To study the effect of a genetic p110γ/δ deficiency on eosinophil numbers, leukocyte suspensions from organs of untreated p110γ^-/-^, p110δ^-/-^, p110γ/δ^-/-^ and wildtype (WT) mice were analysed by flow cytometry (Fig 1A). We found that percentages and total cell counts of eosinophils in bone marrow (BM), spleen, lung, and blood from p110γ/δ^-/-^ mice were significantly elevated not only compared to WT, but also to p110γ^-/-^ or p110δ^-/-^ mice (Fig 1B–1E). Consistently, serum concentrations of the eosinophilic growth factor IL-5 were significantly higher in p110γ/δ^-/-^ mice compared to WT mice, whereas no increase in IL-5 was detectable in p110γ^-/-^ or p110δ^-/-^ mice (Fig 1F).

IgE plays an essential role in type I hypersensitivity, which manifests itself in many allergic diseases including allergic asthma [9]. As increased IgE levels were previously detected in p110γ^KO^/δ^D910A^ mice [23], we measured basal IgE concentrations in the serum of p110γ^-/-^, p110δ^-/-^, p110γ/δ^-/-^ and WT mice (Fig 1G). IgE was elevated 26-fold in p110γ/δ^-/-^ mice in comparison to WT and around 15-fold as compared to single KO animals.

This confirms that the absence of enzymatic activity of PI3Kγ and -δ results in IgE hyperproduction and IL-5-driven eosinophilic inflammation.

### Deficiency in p110γ, p110δ and p110γ/δ reduces bronchoalveolar immune cell infiltration

To study the impact of increased basal levels of eosinophilic granulocytes and IgE, we examined the immune response of p110γ^-/-^, p110δ^-/-^, p110γ/δ^-/-^ and the corresponding WT mice in an OVA-induced allergic airway inflammation model. Mice were sensitised twice with an intraperitoneal injection of a mixture of OVA and adjuvant, followed by a challenge with an aerosolised OVA solution for three consecutive days (S1 Fig). Numbers of infiltrating immune cells in the bronchoalveolar space and lung tissue as well as the degree of bronchial hyperresponsiveness (BHR) served as measures of asthma severity. Due to the complexity of the experimental setup, each KO group was compared to its corresponding WT group. For better comparison between the KO groups, cell counts (S1 Table) were normalised to their corresponding OVA-treated WT group and expressed as cell ratios.

To analyse OVA-specific cell infiltration into the bronchoalveolar space, bronchoalveolar lavage (BAL) was performed to collect the bronchoalveolar lavage fluid (BALF). BALF cell numbers from OVA-treated and PBS-treated p110γ^-/-^, p110δ^-/-^ and p110γ/δ^-/-^ mice and their corresponding WT control groups were determined.

**Fig 2:**
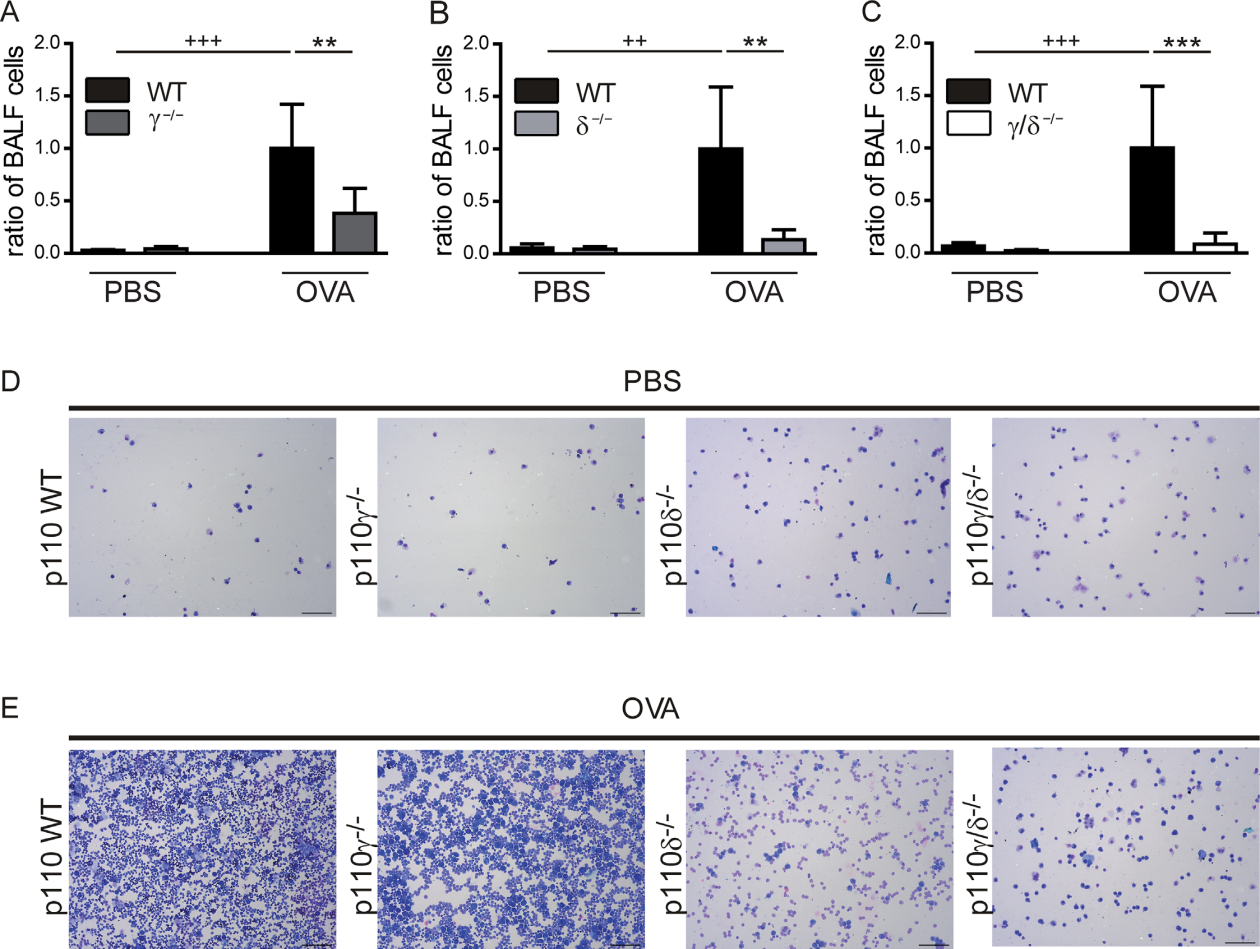
Bronchoalveolar cell infiltration is reduced in OVA-treated p110γ^-/-^, p110δ^-/-^, and p110γ/δ^-/-^ mice. To determine OVA-induced bronchoalveolar cell infiltration, cells were collected by BAL and counted with a Neubauer counting chamber. For better comparison between the knock-out groups, cell counts were normalized to the corresponding OVA WT group. To this end, ratios of BALF cells were calculated as individual cell count / mean cell count of the corresponding OVA WT group. (**A—C**) BALF cells from OVA-treated and PBS-treated KO and corresponding WT mice. **(A**) BALF cells from p110γ^-/-^ and WT mice (n = 3–6). (**B**) BALF cells from p110δ^-/-^ and WT mice (n = 4–5). (**C**) BALF cells from p110γ/δ^-/-^ and WT mice (n = 5–6). Bars express means + SD. Data were analysed by One-way ANOVA followed by Bonferroni’s comparison tests for selected pairs of columns. ^+++^ P < 0.001, ^++^ P < 0.01, ^+^ P < 0.05. ^+^ indicate differences between WT PBS and WT OVA groups. ***P < 0.001, **P < 0.01, *P < 0.05. Asterisks indicate differences between OVA-treated groups. Representative cellspins of BALF samples after (**D**) PBS and (**E**) OVA-treatment. 1/5 of total BAL cells were used for each cell spin. Magnification 100x, scale = 100 μM.

The successful induction of an OVA-specific allergic asthmatic response was verified by significantly increased BALF cell numbers in the OVA-treated WT groups (p110γ^+/+^, p110δ^+/+^ and p110γ/δ^+/+^) as compared to the PBS-treated controls. This demonstrated an OVA-induced cell infiltration into the bronchoalveolar space (Fig 2A–2C). However, this cell infiltration was significantly reduced in OVA-treated p110γ^-/-^, p110δ^-/-^ and p110γ/δ^-/-^ mice compared to the corresponding OVA-treated WT groups (Fig 2A–2C). Of note, this effect was most pronounced in p110γ/δ^-/-^ mice, exhibiting BALF cell numbers that were comparable to PBS-treated p110γ/δ^-/-^ mice (Fig 2C). These findings were corroborated by cellspin analyses of BALF samples from PBS-treated (Fig 2D) and OVA-treated (Fig 2E) p110γ^-/-^, p110δ^-/-^ and p110γ/δ^-/-^ mice. Collectively, these data suggest that deficiency in p110γ, p110δ and p110γ/δ protects against OVA-induced bronchoalveolar immune cell infiltration. To analyse these infiltrated cells in more detail, we next performed differential cell counts in BALF and lung tissue.

### Deficiency in p110γ, p110δ and p110γ/δ reduces bronchoalveolar eosinophil, neutrophil, T and B cell infiltration

**Fig 3:**
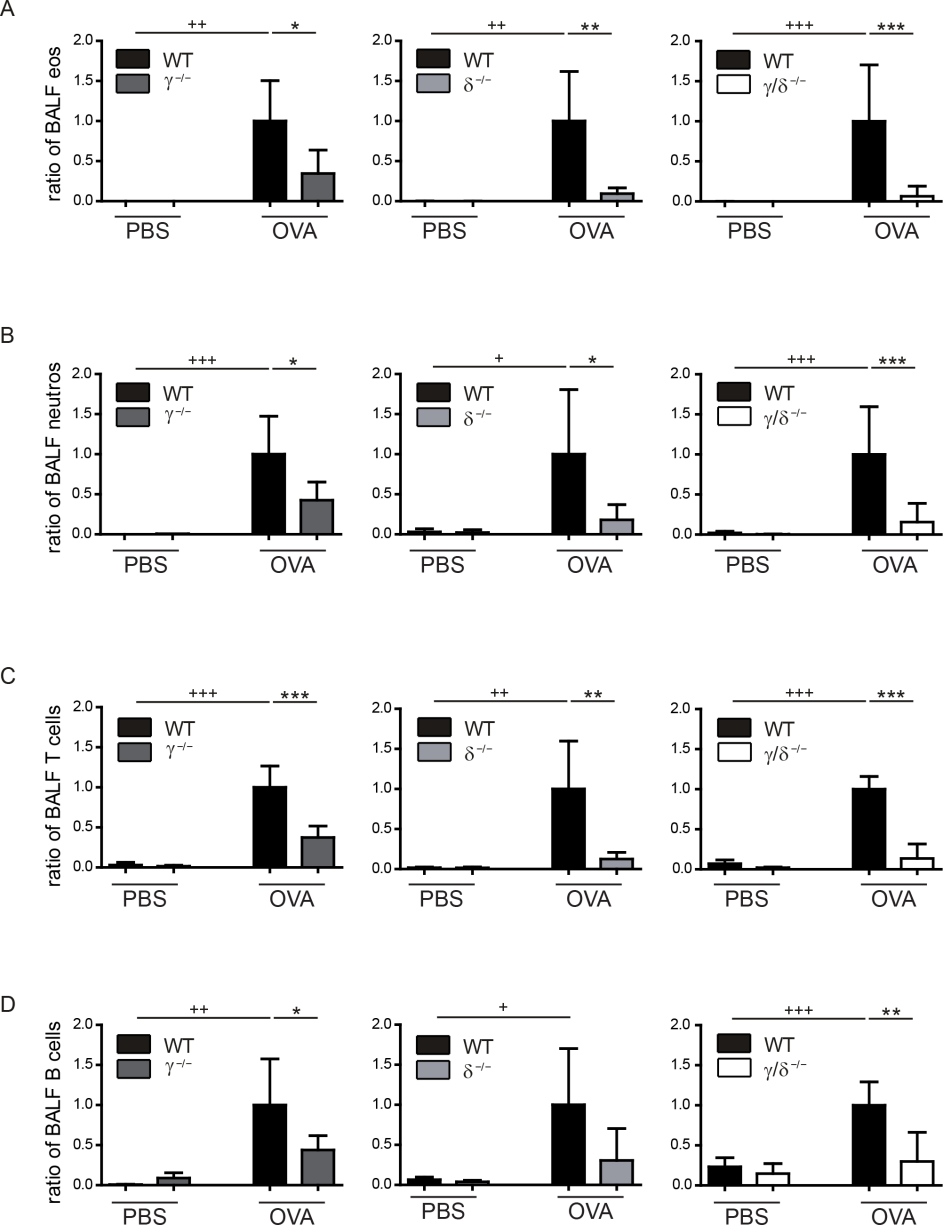
Bronchoalveolar infiltration of eosinophils, neutrophils, T and B cells is reduced in OVA-treated p110γ^-/-^, p110δ^-/-^, and p110γ/δ^-/-^ mice. To determine the number of eosinophils, neutrophils, T and B cells in the BALF from OVA-treated and PBS-treated KO and corresponding WT mice, cells were collected, and analysed by flow cytometry. Cell counts were normalised as described in Fig 2. (**A**) Eosinophils (eos) in BALF from p110γ^-/-^ and WT mice (left), from p110δ^-/-^ and WT mice (middle), and from p110γ/δ^-/-^ and WT mice (right). (**B**) Neutrophils (neutros) in BALF from p110γ^-/-^ and WT mice (left), p110δ^-/-^ and WT mice (middle), and p110γ/δ^-/-^ and WT mice (right). (**C**) T cells in BALF from p110γ^-/-^ and WT mice (left), p110δ^-/-^ and WT mice (middle), and p110γ/δ^-/-^ and WT mice (right). (**D**) B cells in BALF from p110γ^-/-^ and WT mice (left), p110δ^-/-^ and WT mice (middle), and p110γ/δ^-/-^ and WT mice (right). Data (n = 3–6) are presented as means + SD. Data were analysed by One-way ANOVA followed by Bonferroni’s comparison tests for selected pairs of columns. ^+++^ P < 0.001, ^++^ P < 0.01, ^+^ P < 0.05. ^+^ indicate differences between WT PBS and WT OVA groups. ***P < 0.001, **P < 0.01, *P < 0.05. Asterisks indicate differences between OVA-treated groups.

After OVA-challenge, cell counts of eosinophils were significantly increased in BALF of all OVA-treated WT mice in comparison to PBS-treated controls (Fig 3A). Comparisons between the OVA-treated groups demonstrated that bronchoalveolar eosinophil counts were significantly lower in p110γ^-/-^ (Fig 3A, left), p110δ^-/-^ (Fig 3A, middle), and p110γ/δ^-/-^ mice (Fig 3A, right). Moreover, despite the fact that p110γ/δ^-/-^ mice exhibited increased basal eosinophil counts in lung tissue (Fig 1D), the lack of OVA-specific bronchoalveolar eosinophilic infiltration was most pronounced in p110γ/δ^-/-^ mice (Fig 3A, right). Neutrophils are also involved in the onset and progression of allergic asthma [3,4]. Accordingly, bronchoalveolar neutrophil counts were significantly increased in OVA-treated WT groups compared to PBS-treated controls (Fig 3B). Again, OVA-induced bronchoalveolar neutrophil infiltration was significantly reduced in p110γ^-/-^, p110δ^-/-^ and p110γ/δ^-/-^ mice in comparison to WT mice (Fig 3B). Similar to the granulocytes, T and B cells were reduced in all OVA-treated knock-out groups (Fig 3C and 3D).

### Deficiency in p110δ and p110γ/δ reduces lung tissue infiltration by eosinophils, neutrophils, T and B cells

**Fig 4:**
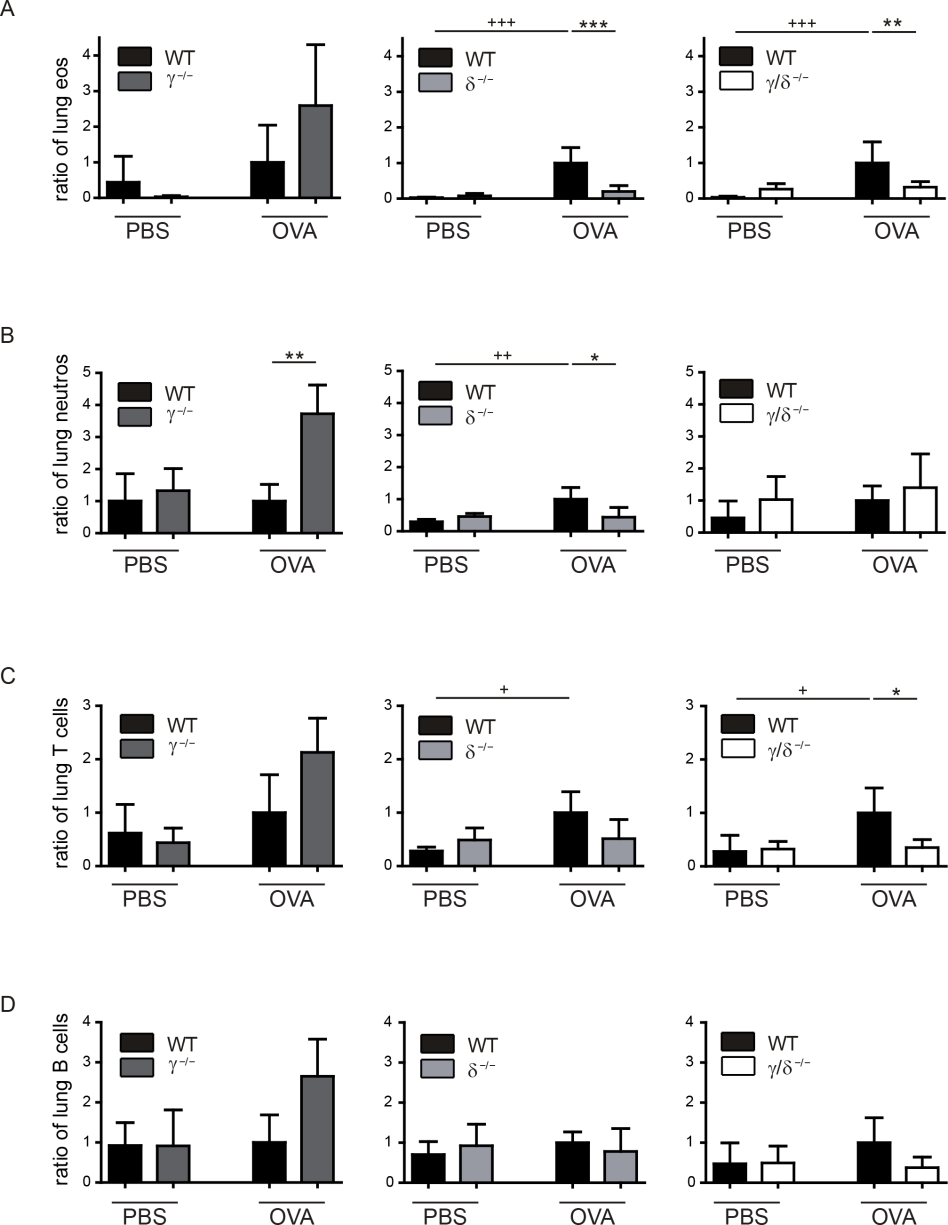
Lung tissue infiltration by eosinophils, neutrophils, T and B cells is only reduced in OVA-treated p110δ^-/-^ and p110γ/δ^-/-^ mice. To determine OVA-induced infiltration of immune cell populations into the lung tissue, leukocytes were prepared from lungs after BAL and exsanguination of PBS-treated and OVA-treated KO and corresponding WT mice. Cell populations were analysed by flow cytometry. Cell counts were normalised as described in Fig 2. (**A**) Eosinophils (eos) in lung tissue from p110γ^-/-^ and WT mice (left), p110δ^-/-^ and WT mice (middle), and p110γ/δ^-/-^ and WT mice (right). (**B**) Neutrophils (neutros) in lung tissue from p110γ^-/-^ and WT mice (left), p110δ^-/-^ and WT mice (middle), and p110γ/δ^-/-^ and WT mice (right). (**C**) T cells in lung tissue from p110γ^-/-^ and WT mice (left), p110δ^-/-^ and WT mice (middle), and p110γ/δ^-/-^ and WT mice (right). (**D**) B cells in lung tissue from p110γ^-/-^ and WT mice (left), p110δ^-/-^ and WT mice (middle), and p110γ/δ^-/-^ and WT mice (right). Data (n = 3–6) are presented as means + SD. Data were analysed by One-way ANOVA followed by Bonferroni’s comparison tests for selected pairs of columns. ^+++^ P < 0.001, ^++^ P < 0.01, ^+^ P < 0.05. ^+^ indicate differences between WT PBS and WT OVA groups. ***P < 0.001, **P < 0.01, *P < 0.05. Asterisks indicate differences between OVA-treated groups.

Immune cell infiltration into the airways requires OVA-induced leukocyte recruitment into the lung, followed by transmigration of cells across endothelial and epithelial barriers. To investigate whether the reduction of immune cells in the BALF in OVA-treated p110γ^-/-^, p110δ^-/-^ and p110γ/δ^-/-^ mice resulted from an impaired leukocyte recruitment into the lung, we determined OVA-induced cell infiltration into the lung tissue. To this end lung cells were prepared following BAL and exsanguination of the animals and then analysed by flow cytometry. We found that OVA-treated p110γ^-/-^ mice showed a trend towards an increased infiltration of eosinophils into the lung tissue when compared to the corresponding WT group (Fig 4A, left). In contrast, eosinophil numbers in the lung tissue of OVA-treated p110δ^-/-^ mice were significantly lower (Fig 4A middle). As expected from the analysis of the basal eosinophil numbers in untreated p110γ/δ^-/-^ mice (Fig 1D), we also found an increase of eosinophils in the lung tissue of PBS-treated p110γ/δ^-/-^ mice, which was not altered after OVA-treatment (Fig 4A, right). Rather, eosinophil numbers in the lung tissue of OVA-treated p110γ/δ^-/-^ mice were still significantly lower as compared to OVA-treated WT mice.

When analysing neutrophils in the lung tissue, similar, but not identical, infiltration patterns were observed: while these cells were significantly increased in lungs of OVA-treated p110γ^-/-^ mice (Fig 4B, left), a significant reduction in OVA-treated p110δ^-/-^ mice was evident (Fig 4B, middle), whereas there were no significant differences between PBS- and OVA-treated p110γ/δ^-/-^ mice (Fig 4B, right).

T and B cells in the lung tissue of OVA-treated p110γ^-/-^ or p110δ^-/-^ mice showed no significant differences compared to the corresponding WT mice (Fig 4C and 4D, left, middle). However, in p110γ^-/-^ mice T and B cell numbers tended to be increased after OVA-treatment (Fig 4C and 4D, left). Moreover, T cell (Fig 4C, right) and B cell numbers (Fig 4D, right) in p110γ/δ^-/-^ mice were lower, when compared to WT controls.

Therefore, in p110δ^-/-^ and p110γ/δ^-/-^ mice, but not in p110γ^-/-^ mice, the infiltration pattern seen in the bronchoalveolar space was roughly mirrored in the lung tissue. This might indicate that in our experimental model the immune cells of OVA-treated p110γ^-/-^ mice are recruited into the lung, but partly fail to transmigrate into the airways. Collectively, this suggests that p110γ and p110δ are both involved in allergen-induced recruitment of eosinophils into the lungs, although the observed differences in the lung tissue infiltration of eosinophils between p110γ^-/-^ and p110δ^-/-^ mice suggest that the two isoforms play distinct roles in the migration and transmigration process.

### OVA-specific IgE is increased in p110γ/δ^-/-^ mice during allergic airway inflammation

**Fig 5:**
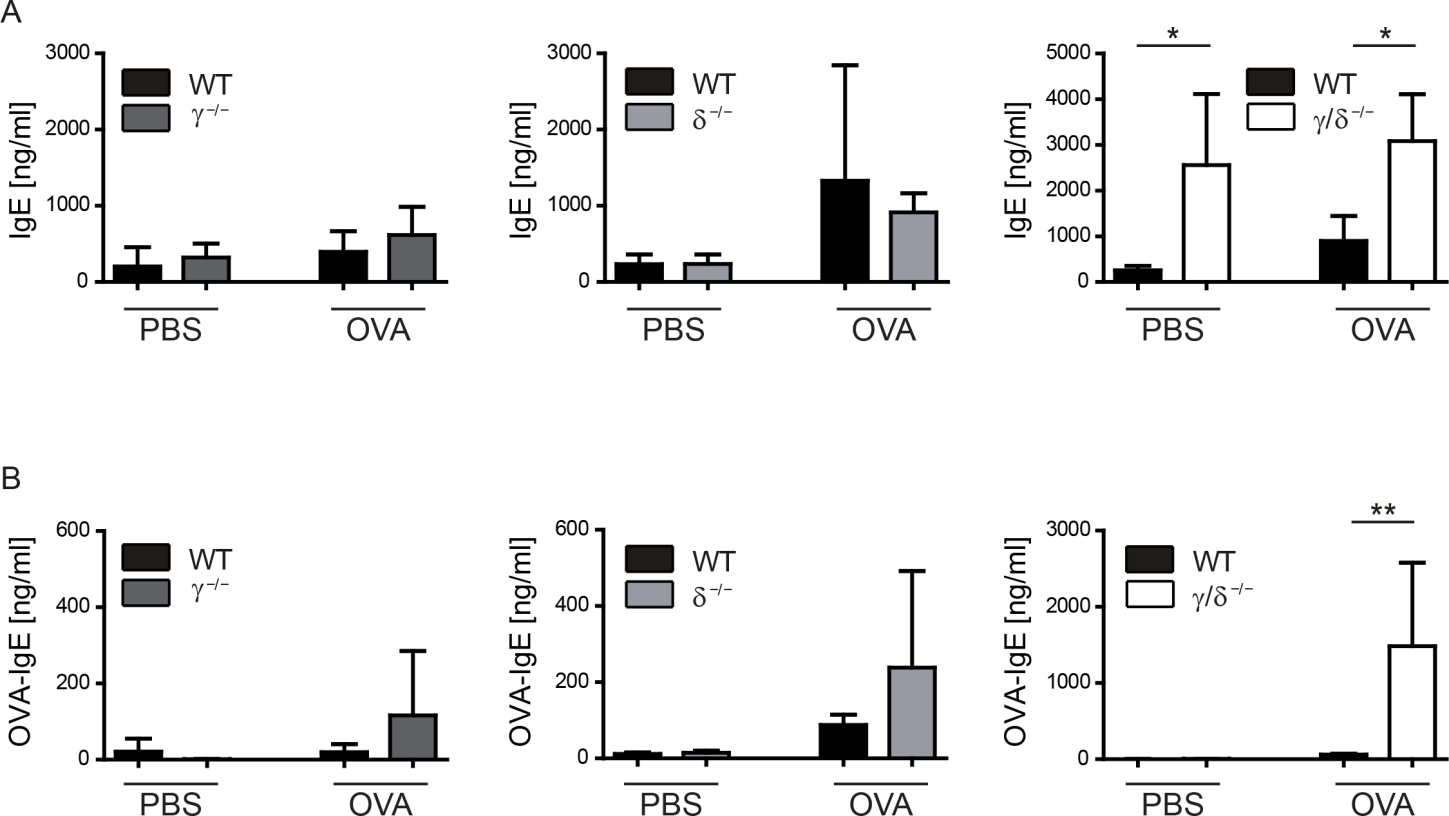
Basal and OVA-specific IgE levels are increased in p110γ/δ^-/-^ mice. Total and OVA-specific IgE was determined in sera of PBS-treated and OVA-treated KO and corresponding WT mice using ELISA. (**A**) Total serum IgE. (**B**) OVA-specific serum IgE. Bars express means + SD; n = 4–8 pooled from three independent experiments. Data were analysed by One-way ANOVA followed by Bonferroni’s comparison tests for selected pairs of columns; **P < 0.01, *P < 0.05.

IgE has an essential role in allergic asthma [9]. As untreated p110γ/δ^-/-^ mice exhibited elevated total IgE concentrations (Fig 1G), we determined total and OVA-specific IgE during OVA-induced allergic asthma (Fig 5). Interestingly, OVA-treatment did not further increase the inherently elevated total IgE levels in p110γ/δ^-/-^ mice, and the levels were still significantly higher than in OVA-treated WT mice (Fig 5A, right). In OVA-treated p110γ^-/-^ or p110δ^-/-^ mice, we could not detect any significant differences in total IgE concentrations (Fig 5A, left and middle). In contrast to the single KO groups, p110γ/δ^-/-^ mice showed a massive increase in OVA-specific IgE (Fig 5B). This suggests that double-deficiency in p110γ/δ increases total IgE concentrations at the basal state and enhances the production of allergen-specific IgE during OVA-induced allergic airway inflammation.

### Bronchial hyperresponsiveness and goblet cell metaplasia are reduced in OVA-treated p110γ/δ^-/-^ mice

**Fig 6:**
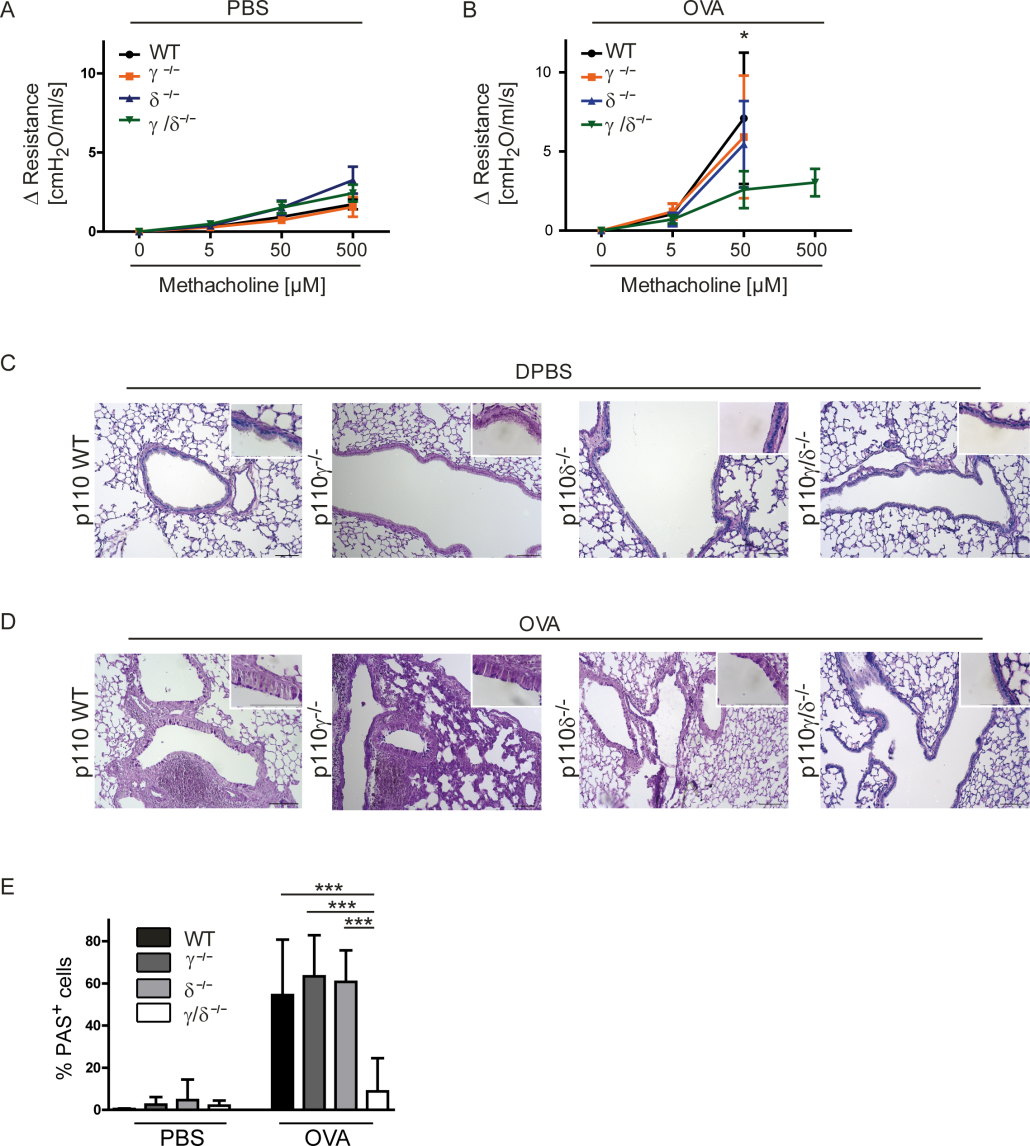
Bronchial hyperresponsiveness and goblet cell metaplasia are reduced in OVA-treated p110γ/δ^-/-^ mice. To determine bronchial hyperresponsiveness, lung function analysis was performed using the IPL and changes in airway resistance were measured following systemic application of rising doses of methacholine (MCh). Some values had to be excluded, e.g. when lungs were damaged during the experiments. Changes in airway resistance in (**A**) PBS-treated (n = 3–10) and (**B**) OVA-treated (n = 5–7) KO and WT mouse groups. All three WT groups were analyzed and pooled for a clearer graphical presentation. Data in (**B**) were analysed by Two-way ANOVA followed by Bonferroni’s comparison tests *P < 0.05. (**C, D**) Mucus production in PBS-treated and in OVA-treated KO and WT mice. To measure mucus production, lungs were collected after IPL and cut into 6 μm thick slices. Sections were stained for carbohydrates using the periodic acid-Schiff (PAS) reaction and counter stained with H&E. Representative lung tissue sections from WT, p110γ^-/-^, p110δ^-/-^, and p110γ/δ^-/-^ mice after (**C**) PBS-treatment and (**D**) OVA-treatment. Magnification 100x, inserts 630x. (**E**) PAS^+^ cells (pink) per basement membrane in mm. Bars express means + SD; Data (n = 3–6 mice) were analysed by One-way ANOVA followed by Tukey’s Multiple Comparison Test; ***P < 0.001.

Asthma is characterized by variable and reversible obstruction of the airflow caused by increased mucus production and bronchoconstriction [4]. This bronchial hyperresponsiveness (BHR) is even inducible by unspecific stimuli, such as the muscarinic agonist methacholine (MCh). The degree of BHR was determined as an additional parameter of severity of OVA-induced airway inflammation. To this end, the isolated, ventilated and perfused lung (IPL) model was used and the muscarinic agonist MCh was applied in increasing concentrations [34]. The airway resistance was measured as an indicator of airway constriction. All PBS-treated KO groups (p110γ^-/-^, p110δ^-/-^, p110γ/δ^-/-^) showed no significant differences in their airway resistance as compared to WT controls (Fig 6A). As expected, the airway resistance massively increased in OVA-treated WT mice (Fig 6B), where measurements were only possible up to a MCh concentration of 50 μM and higher concentrations led to a complete block of airflow. Similar responses were observed in OVA-treated p110γ^-/-^ and p110δ^-/-^ mice. In contrast, OVA-treated p110γ/δ^-/-^ mice showed a significantly lower airway resistance at 50 μM MCh and 50% of the mice could be measured up to the highest MCh concentration (500 μM), which otherwise was only possible for PBS-treated mice (Fig 6B).

Mucus is an important contributor to airway obstruction in asthma, and increased numbers of mucus-secreting goblet cells are commonly seen in the lung epithelium of human asthmatic patients [36], but also in mouse models of asthma [34]. To determine the number of mucus-containing goblet cells in the airways, lungs were collected after IPL and lung sections were stained for carbohydrates using the periodic acid-Schiff (PAS) reaction. In lung sections of PBS-treated KO or WT mice no differences in the number of PAS^+^ cells were observed (representative images; Fig 6C). In accordance with the findings on airway resistance, OVA-treatment resulted in increased percentages of PAS^+^ cells in WT, p110γ^-/-^ and p110δ^-/-^ mice. In contrast, the analysed lung sections from OVA-treated p110γ/δ^-/-^ mice showed no increase in PAS^+^ cells when compared to the control sections from PBS-treated mice (representative images; Fig 6D). Accordingly, statistical analysis of the lung tissue sections showed a significantly lower numbers of PAS^+^ cells per mm basement membrane in OVA-treated p110γ/δ^-/-^ mice in comparison to the other OVA-treated groups (Fig 6E). Thus, p110γ/δ double deficiency partly protects from allergen-induced BHR.

## Discussion

In this study we demonstrated that mice lacking both p110γ and δ exhibit increased eosinophil counts and elevated serum IL-5 and IgE levels in a basal state, suggesting that a deficiency of p110γ/δ causes IgE-hyperproduction and eosinophilia. In spite of the fact that eosinophilia and increased serum IgE levels are two key elements of allergic asthma, p110γ/δ^-/-^ mice were partly protected against OVA-induced experimental airway inflammation.

IgE plays a major role in the development of allergen-induced chronic airway inflammation. Cross-linking of IgE-FcεRI complexes by allergens leads to cellular degranulation and the release of cytokines, histamine and lipid mediators [9,10,37]. These factors are known to be involved in the recruitment of Th2 cells, eosinophils, basophils, as well as in vasodilatation and bronchoconstriction [37]. Monomeric IgE has been shown to increase mast cell survival and induce cytokine secretion in the absence of cross-linking by allergens [9,10]. Thus, one might speculate that increased levels of natural IgE in a basal state predispose p110γ/δ mice to aggravated allergic responses.

Our results demonstrate, for the first time, that in addition to elevated basal levels of natural IgE p110γ/δ^-/-^ mice exhibit increased antigen-specific IgE concentrations following OVA-treatment. The role of p110γ and p110δ signalling in allergen-induced production of OVA-specific IgE is unclear. On one hand, the genetic deficiency in p110γ had no influences on allergen-induced production of OVA-specific IgE [12]. On the other hand, pharmacological inhibition of p110δ with IC87114 attenuated OVA-induced increases in total and OVA-specific IgE [11]. By contrast, another study found that genetic or pharmaceutical inactivation of p110δ resulted in increases of total and OVA-specific IgE [38]. Our findings, showing that only p110γ/δ^-/-^ mice, but not the single KO groups, exhibited a massive increase in OVA-specific IgE, suggest that both p110γ and p110δ contribute to the regulation of allergen-specific IgE production in these animals.

Although the serum levels of both natural and OVA-specific IgE were drastically elevated in OVA-treated p110γ/δ^-/-^ mice, this did not result in an exacerbated phenotype of allergic airway inflammation, in fact it inferred protection from the disease syndrome. Impaired allergic responses in the presence of increased IgE concentrations may be due to altered mast cell numbers and/or reduced IgE-signalling. It has been previously reported that genetic inactivation of p110δ decreases mast cell numbers in mice [39]. Furthermore, both p110γ and p110δ appear to be required for IgE/antigen-triggered allergic responses, with PI3Kδ acting earlier and PI3Kγ acting later in response to IgE [17].

Allergic asthma is also characterized by the accumulation of eosinophils in the lungs [40], and increasing eosinophil numbers in humans correlate with the disease severity of asthma [8]. Reduction of eosinophils by application of anti-IL-5 mAbs ameliorated asthma in a selected sub-population of asthma patients with demonstrable eosinophilic airway inflammation (references in Walsh *et*. *al*. [6]). Decreased numbers of eosinophils in anti-IL-5-treated mice were protective in an OVA-model of allergic asthma (references in Walsh *et*. *al*. [6]). Asthma studies using two strains of eosinophil-deficient mice demonstrated that eosinophils play a significant role in asthma-related airway hyperresponsiveness and mucus accumulation [41]. In C57BL/6 mice eosinophils are required for the induction of OVA-induced asthma [6,7].

Our findings demonstrated that, at a basal state, eosinophilia in p110γ/δ^-/-^ mice correlated with increased levels of IL-5. This indicates that in p110γ/δ^-/-^ mice basal eosinophilia is driven by type 2 immune responses. Following OVA-treatment, eosinophil numbers in the lung tissue of p110γ/δ^-/-^ mice were not further increased and the animals were protected against eosinophilic infiltration into the airways. Similarly, p110δ^-/-^ mice exhibited a reduced OVA-related lung tissue migration and airway infiltration of eosinophils compared to WT controls. In contrast, in p110γ^-/-^ mice numbers of OVA-induced lung tissue eosinophils exceeded WT levels, although the number of eosinophils infiltrating into the airways was significantly reduced. Other studies confirm the involvement of p110γ and p110δ in eosinophil migration [13,14,21]. The fact that the increased basal levels of eosinophils in the lungs of p110γ/δ^-/-^ mice did not promote airway inflammation following OVA-treatment might be explained by an impaired allergen-induced cell activation and degranulation of p110γ/δ^-/-^ eosinophils. However, so far the importance of PI3K signalling in eosinophil activation has only been shown by pan-PI3K inhibition using wortmannin [42,43].

The striking phenotype of p110γ/δ^-/-^ mice exhibiting a Th2-driven eosinophilia and high IgE levels might be explained by an impaired TCR-activation-mediated Ca^2+^ signalling [44] and consecutively impaired NFAT activation [45]. Alternatively, the phenotype might be related to T cell lymphopenia in p110γ/δ^-/-^ mice [29,30]. Low T cell counts are additionally observed in p110γ^KO^δ^D910A^ mice, which also suffer from IgE-hyperproduction and eosinophilia [23]. Although the mechanism is unclear, the correlation between low T cell numbers and type 2 cytokines, eosinophilia and/or IgE hyperproduction appears to be a common phenomenon in humans [32,46] and mice [33].

Collectively, this may suggest that eosinophilia and IgE-hyperproduction in p110γ/δ^-/-^ mice result from a lymphopenia-driven induction of Th2 responses. If so, then this may have serious consequences for the treatment with dual pharmacological inhibitors of p110γ and p110δ. This is supported by the finding that, similar to genetic ablation, pharmacological inactivation of p110γ and p110δ with the dual-specific inhibitor CAL-130 inhibits T cell development [31]. This suggests that long-term treatment of patients with pharmacological inhibitors of p110γ and p110δ could induce lymphopenia, possibly accompanied by induction of Th2 responses, increased IgE levels and eosinophilia.

In summary, our data provide further evidence for the importance of combined p110γ and p110δ signalling in pathogenesis of allergic airway inflammation, supporting the concept that short-term treatment with dual pharmacological inhibitors of p110γ/δ might ameliorate the severity of the disease syndrome. Nevertheless, the immunocompromised phenotype of p110γ/δ^-/-^ mice suggests that these inhibitors could harbour severe side effects if applied in long-term treatment in patients, causing severe immune defects and thereby possibly also increasing the susceptibility towards opportunistic infections.

## Supporting Information

S1 Fig Timeline of OVA-dependent induction of allergic airway inflammation. Asthma was induced as described by Park, *et al*. 2010 [14]. Mice were sensitised on day 1 and 14 with an i.p. injection of OVA in Imject^TM^ Alum, followed by a challenge with aerosolised OVA for three consecutive days (days 21–23). On day 25 animals were sacrificed and analysed, either by collecting the BALF and lung tissue, or by performing lung function analysis with the IPL. Control animals were sensitised with Imject^TM^ Alum only and challenged with PBS.
(EPS)

S2 Fig Gating strategy for BALF leukocytes. T and B cells were gated as CD11b^-^ SSC^low^ singlet cells that were CD3ε^+^ or CD19^+^, respectively (upper and third lane, far right graphs). Neutrophils were identified following exclusion of CD11b^-^ SSC^low^ cells ("not gate") and consecutive gating on CD11b^+^ Ly6G^+^ cells (second and forth lane, left graphs). After exclusion of CD11b^+^ Ly6G^+^ neutrophils ("not gate") eosinophils were gated as F4/80^low/-^ Siglec F^+^ cells (second and forth lane, right graphs). Shown are representative samples from one OVA-treated WT and one OVA-treated p110γ/δ^-/-^ mouse.
(EPS)

S1 Table Absolute Numbers of BALF and lung tissue cells. Absolute numbers of BALF and lung tissue cells from Figs 3 and 4.
(DOCX)
