# Correction: p110γ/δ Double-Deficiency Induces Eosinophilia and IgE Production but Protects from OVA-Induced Airway Inflammation

**DOI:** 10.1371/journal.pone.0163577

**Published:** 2016-09-19

**Authors:** Benedikt Mothes, Kirsten Bucher, Susanne Ammon-Treiber, Matthias Schwab, Roland P. Piekorz, Emilio Hirsch, Bernd Nürnberg, Sandra Beer-Hammer

The published article contains an earlier version of Fig 6, which does not reflect revisions made during the review process. Please see the correct Fig 6 and its legend here.

**Fig 6:**
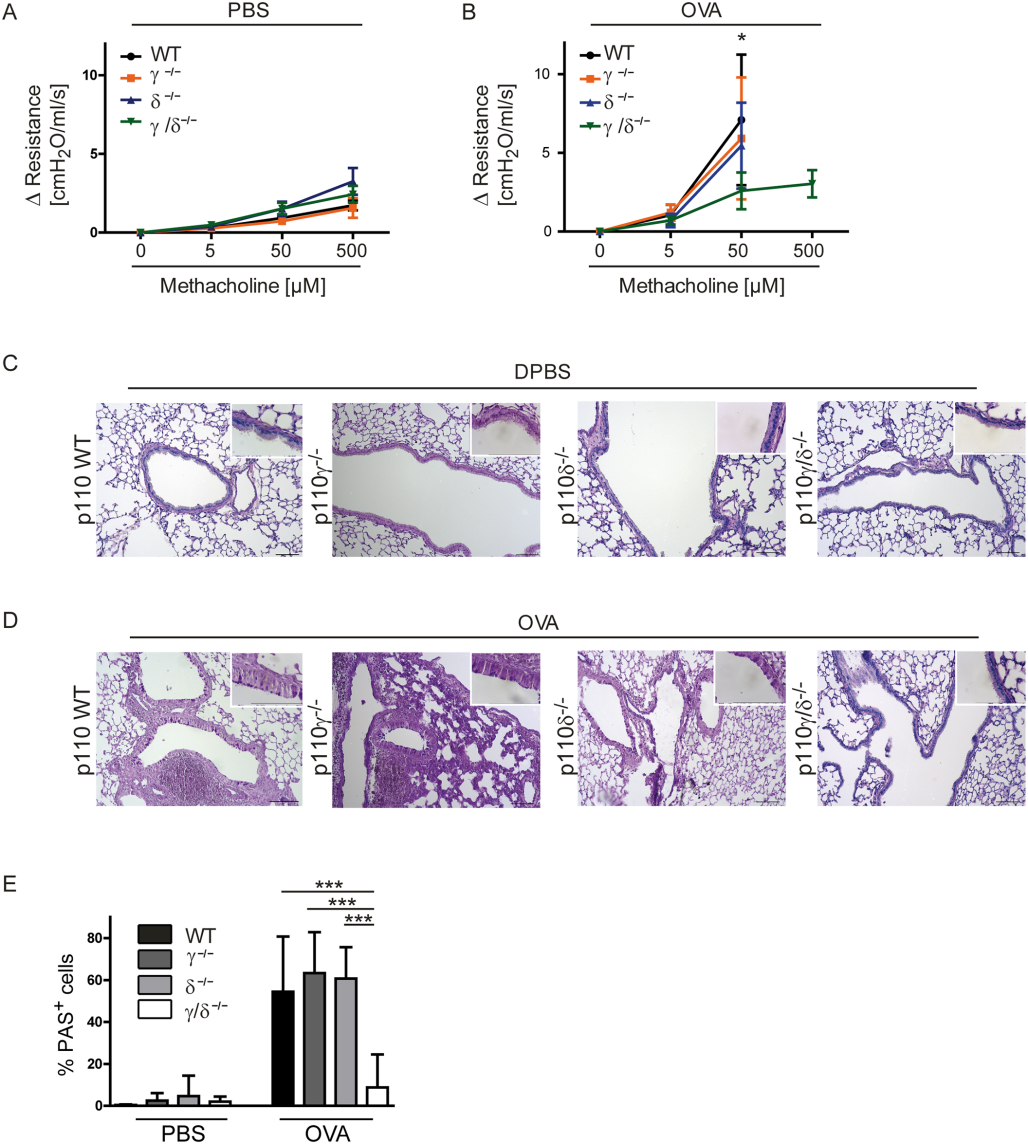
Bronchial hyperresponsiveness and goblet cell metaplasia are reduced in OVA-treated p110γ/δ^-/-^ mice. To determine bronchial hyperresponsiveness, lung function analysis was performed using the IPL and changes in airway resistance were measured following systemic application of rising doses of methacholine (MCh). Some values had to be excluded, e.g. when lungs were damaged during the experiments. Changes in airway resistance in (A) PBS-treated (n = 3–10) and (B) OVA-treated (n = 5–7) KO and WT mouse groups. All three WT groups were analyzed and pooled for a clearer graphical presentation. Data in (B) were analysed by Two-way ANOVA followed by Bonferroni’s comparison tests *P < 0.05. (C, D) Mucus production in PBS-treated and in OVA-treated KO and WT mice. To measure mucus production, lungs were collected after IPL and cut into 6 μm thick slices. Sections were stained for carbohydrates using the periodic acid-Schiff (PAS) reaction and counter stained with H&E. Representative lung tissue sections from WT, p110γ^-/-^, p110δ^-/-^, and p110γ/δ^-/-^ mice after (C) PBS-treatment and (D) OVA-treatment. Magnification 100x, inserts 630x. (E) PAS^+^ cells (pink) per basement membrane in mm. Bars express means + SD; Data (n = 3–6 mice) were analysed by One-way ANOVA followed by Tukey’s Multiple Comparison Test; ***P < 0.001.
