# Supplementary material for: p110γ/δ Double-Deficiency Induces Eosinophilia and IgE Production but Protects from OVA-Induced Airway Inflammation
Source: PLoS One. 2016 Jul 21;11(7):e0159310. doi: 10.1371/journal.pone.0159310 (PMC4956235; doi:10.1371/journal.pone.0159310)
Supplement: S1 Table — Absolute numbers of BALF and lung tissue cells from Figs 3 and 4. (DOCX) [file pone.0159310.s003.docx]

**Supplemental Table 1**

| [x 10^4] | | | WT (γ^+/+^) PBS | | γ^-/-^  PBS | | | WT (γ^+/+^) OVA | γ^-/-^  OVA | WT (δ^+/+^) PBS | δ^-/-^  PBS | WT (δ^+/+^) OVA | δ^-/-^  OVA | WT (γ/δ^+/+^) PBS | γ/δ^-/-^  PBS | WT (γ/δ^+/+^) OVA | γ/δ^-/-^  OVA |
| --- | --- | --- | --- | --- | --- | --- | --- | --- | --- | --- | --- | --- | --- | --- | --- | --- | --- |
| **BALF** |  | |  | |  | | |  |  |  |  |  |  |  |  |  |  |
| **total cells** | mean | | 5.00 | | 7.54 | | | 174.30 | 66.37 | 7.81 | 6.06 | 137.10 | 18.69 | 6.15 | 1.98 | 93.39 | 7.73 |
|  | SD | | 1.30 | | 3.95 | | | 73.23 | 41.45 | 4.98 | 3.42 | 80.87 | 12.94 | 3.03 | 1.09 | 54.96 | 10.02 |
| Eosinophils | mean | | 0.03 | | 0.05 | | | 135.00 | 46.63 | 0.14 | 0.08 | 106.20 | 9.92 | 0.10 | 0.04 | 73.77 | 4.08 |
|  | SD | | 0.02 | | 0.03 | | | 68.16 | 39.56 | 0.12 | 0.08 | 65.76 | 7.77 | 0.03 | 0.02 | 45.40 | 7.94 |
| Neutrophils | mean | | 0.00 | | 0.04 | | | 4.76 | 2.03 | 0.11 | 0.08 | 3.63 | 0.66 | 0.03 | 0.01 | 2.20 | 0.23 |
|  | SD | | 0.00 | | 0.03 | | | 2.26 | 1.08 | 0.14 | 0.13 | 2.93 | 0.69 | 0.03 | 0.00 | 1.76 | 0.35 |
| T cells | mean | | 0.34 | | 0.18 | | | 11.11 | 4.16 | 0.20 | 0.18 | 11.12 | 1.39 | 0.25 | 0.07 | 5.80 | 0.47 |
|  | SD | | 0.36 | | 0.13 | | | 2.94 | 1.59 | 0.09 | 0.12 | 6.65 | 0.94 | 0.16 | 0.03 | 5.20 | 0.63 |
| B cells | mean | | 0.01 | | 0.12 | | | 1.35 | 0.59 | 0.05 | 0.03 | 0.81 | 0.25 | 0.04 | 0.03 | 0.47 | 0.06 |
|  | SD | | 0.00 | | 0.09 | | | 0.77 | 0.24 | 0.03 | 0.01 | 0.57 | 0.32 | 0.02 | 0.02 | 0.64 | 0.07 |
| **Lung tissue** |  | |  | |  | | |  |  |  |  |  |  |  |  |  |  |
| Eosinophils | mean | | 122.30 | | 9.29 | | | 277.80 | 720.50 | 13.14 | 33.68 | 448.50 | 89.27 | 10.29 | 78.67 | 295.90 | 95.22 |
|  | SD | | 202.00 | | 8.46 | | | 289.80 | 475.10 | 5.49 | 30.69 | 194.40 | 74.36 | 10.74 | 45.23 | 174.80 | 45.24 |
| Neutrophils | mean | | 32.45 | | 42.81 | | | 32.21 | 120.00 | 33.14 | 51.51 | 112.00 | 49.31 | 27.77 | 61.88 | 60.11 | 84.23 |
|  | SD | | 27.31 | | 22.04 | | | 16.83 | 28.91 | 8.42 | 11.01 | 40.82 | 33.94 | 31.35 | 43.41 | 27.49 | 63.44 |
| T cells | mean | | 9.97 | | 7.08 | | | 16.14 | 34.38 | 7.18 | 12.38 | 25.30 | 12.99 | 4.42 | 5.10 | 15.81 | 5.57 |
|  | SD | | 8.67 | | 4.40 | | | 11.47 | 10.31 | 1.82 | 5.68 | 9.89 | 9.04 | 4.77 | 2.26 | 7.36 | 2.35 |
| B cells | mean | | 13.06 | | 12.93 | | | 14.11 | 37.43 | 17.05 | 22.38 | 24.20 | 18.88 | 8.17 | 8.51 | 17.21 | 6.50 |
|  | SD | | 8.04 | | 12.66 | | | 9.71 | 13.08 | 7.83 | 12.97 | 6.52 | 13.92 | 8.94 | 7.22 | 10.67 | 4.56 |
|  | | | | | |  |  |  |  |  |  |  |  |  |  |  |  |
|  | |  | |  | | |  |  |  |  |  |  |  |  |  |  |  |
